# Pharmacotherapy for chronic obesity management: a look into the future

**DOI:** 10.1007/s11739-023-03237-4

**Published:** 2023-05-30

**Authors:** Mariana Abdel-Malek, Lisa Yang, Alexander Dimitri Miras

**Affiliations:** 1 Imperial College Healthcare NHS Trust, London, UK; 2 School of Medicine, Ulster University, Derry~Londonderry, UK; 3 Department of Metabolism, Digestion and Reproduction, Imperial College London, London, UK

**Keywords:** Obesity, Glucagon-like peptide 1, Glucagon dependent insulinotropic polypeptide, Type 2 diabetes, Tirzepatide, Amylin

## Abstract

Substantial leaps have been made in the drug discovery front in tackling the growing pandemic of obesity and its metabolic co-morbidities. Greater mechanistic insight and understanding of the gut-brain molecular pathways at play have enabled the pursuit of novel therapeutic agents that possess increasingly efficacious weight-lowering potential whilst remaining safe and tolerable for clinical use. In the wake of glucagon-like peptide 1 (GLP-1) based therapy, we look at recent advances in gut hormone biology that have fermented the development of next generation pharmacotherapy in diabesity that harness synergistic potential. In this paper, we review the latest data from the SURPASS and SURMOUNT clinical trials for the novel ‘twincretin’, known as Tirzepatide, which has demonstrated sizeable body weight reduction as well as glycaemic efficacy. We also provide an overview of amylin-based combination strategies and other emerging therapies in the pipeline that are similarly providing great promise for the future of chronic management of obesity.

## Introduction

The frontier of obesity pharmacotherapy has been rapidly expanding. Although evolving research pertaining to incretin biology has primarily focussed on diabetes management, the concurrent anti-obesity effects and tolerability observed with pharmacological intervention has enabled pursuit of drug discovery. With diabesity on the rise, the decision-making process for glucose-lowering therapy has evolved to consider adjunctive BMI as well as co-existing cardiovascular or renal disease in its multifaceted treatment. Hence glucagon-like peptide 1 (GLP-1) therapy is now a well-established class in the treatment paradigm for type 2 diabetes (T2DM) given its glycaemic efficacy and weight loss potency as well as favourable cardiometabolic outcomes. This review aims to encompass the latest trial findings of novel agents and emerging therapies with a focus on the alternative neuroendocrine mechanisms that are paving a bright future for pharmacological adjuncts in the management of obesity.

## Background

Liraglutide 3 mg was the first GLP-1 receptor agonist to gain approval at a dosage which was almost double that used for the treatment of T2DM [1]. However, Semaglutide 1 mg once weekly is currently regarded as the most potent GLP-1 mimetic with results of 1.5–1.8% reduction in HbA1c (vs < 0.1–0.4% with placebo) and 4.5–6.5 kg weight loss (vs 1.0–1.4 kg placebo) in the SUSTAIN 1–5 and 7 trials [2]. Semaglutide was further evaluated at the higher dose of 2.4 mg once weekly in the STEP clinical trials programme with primary endpoints specifically for weight loss outcomes in the obese or overweight population and irrespective of concurrent diabetes. Table 1 summarises the anti-obesity efficacy of Semaglutide 2.4 mg in the STEP clinical trials (STEP 1–6, STEP 8). In all the trials, Semaglutide 2.4 mg demonstrated superiority with a higher proportion of patients achieving greater percentage weight loss relative to the comparator (placebo, Liraglutide 3.0 mg, Semaglutide 1.7 mg or 1.0 mg) [3–9]. At 68 weeks, Semaglutide 2.4 mg consistently led to a significant mean reduction in body weight of 14.9% (STEP 1), 16.0% (STEP 3) and 15.8% (STEP 8) which was notably greater than the placebo groups and more than twice that of Liraglutide 3.0 mg (STEP 8; 6.4%) [3, 5, 9]. This substantial weight loss was sustainable at 2 years, as demonstrated in the STEP 5 trial with a mean body weight loss of 15.2% at week 104 in the Semaglutide-treated group (vs 2.4% placebo) [7].

**Table 1:** Summary of findings from the STEP Clinical Trials testing once weekly injectable Semaglutide 2.4 mg for weight loss

Trial	Comparator Group	Participants	Mean change in BW from baseline				Proportion achieving BW loss (%) of					
			Semaglutide 2.4 mg		Comparator		Semaglutide 2.4 mg			Comparator		
			kg	%	kg	%	≥ 5%	≥ 10%	≥ 15%	≥ 5%	≥ 10%	≥ 15%
STEP 1 (*n* = 1961) 68 weeks [3]	Placebo	BMI ≥ 30 kg/m^2^ (or ≥ 27 with ≥ 1 weight-related comorbidity), and with no diabetes	− 15.3	− 14.9	− 2.6	− 2.4	86.4	69.1	50.5	31.5	12.0	4.9
STEP 2 (*n* = 1210) 68 weeks [4]	Semaglutide 1.0 mg	BMI ≥ 27 kg/m^2^ HbA1c 7–10% (53–86 mmol/mol), and diagnosed with T2DM	− 9.7	− 9.64	− 6.9	− 6.99	68.8	45.6	25.8	57.1	28.7	13.7
	Placebo				− 3.5	− 3.42				28.5	8.2	3,2
STEP 3 (*n* = 611) 68 weeks [5]	Placebo	BMI ≥ 30 kg/m^2^ (or ≥ 27 with ≥ 1 weight-related comorbidity), and with no diabetes	− 16.8	− 16.0	− 6.2	− 5.7	86.6	75.3	55.8	47.6	27.0	13.2
STEP 4 (*n* = 902) 68 weeks [6]	Placebo between week 20 to 68 (all received Semaglutide for first 20 weeks)	BMI ≥ 30 kg/m^2^ (or ≥ 27 with ≥ 1 weight-related comorbidity), and with no diabetes		− 17.4		− 5.0	88.7	79.0	63.7	47.6	20.4	9.2
STEP 5 (*n* = 304) 2 years [7]	Placebo	BMI ≥ 30 kg/m^2^ (or ≥ 27 with ≥ 1 weight-related comorbidity), and with no diabetes	− 16.2	− 15.2	− 3.2	− 2.6	77.1	61.8	52.1	34.4	13.3	7.0
STEP 6 (*n* = 401) 68 weeks [8]	Semaglutide 1.7 mg	BMI ≥ 27 kg/m^2^ with ≥ 2 comorbidity or ≥ 30 with ≥ 3 comorbidity (Japan and South Korea)	− 11.5	− 13.2	− 8.19	− 9.6	83	61	41	72	42	24
	Placebo				− 1.70	− 2.1				21	5	3
STEP 8 (*n* = 338) 68 weeks [9]	Liraglutide 3.0 mg daily	BMI ≥ 30 kg/m^2^ (or ≥ 27 with ≥ 1 weight-related comorbidity), and with no diabetes	− 15.3	− 15.8	− 6.8	− 6.4	87.2	70.9	55.6	58.1	25.6	12.0

*BMI* Body Mass Index, *BW* Body Weight, *HbA1c* Haemoglobin A1c

Like GLP-1, glucose-dependent insulinotropic polypeptide (GIP) is an incretin hormone with a postprandial effect on insulin response that is contingent on the modulation of blood glucose and secreted in response to nutrient intake [10]. Given the evolution of GLP-1 receptor agonist drugs, the pharmacological harnessing of this ‘incretin effect’ that underpins meal-stimulated insulin secretion is an avenue that continues to be explored in the treatment of T2DM and obesity. Preclinical studies have illustrated that co-administration of these principal incretin hormones, GLP-1 with GIP, has a synergistic benefit on the insulinotropic as well as glucagonostatic response and thereby additive therapeutic potential for targeting causality in diabesity [11]. These findings have altogether paved the development of a novel GIP/GLP-1 receptor agonist, Tirzepatide, known as a ‘twincretin’ that has shown potent glycaemic efficacy and remarkable weight loss benefit through dual metabolic action.

Furthermore, amylin is a neuroendocrine peptide co-secreted with insulin by pancreatic β cells and acts to inhibit glucagon secretion, slow gastric emptying, and centrally induce satiety. Mechanistic insight into the behaviour of this pancreatic β cell hormone in satiation signalling and weight gain suppression has paved yet another promising pharmacological target [12]. Cagrilintide, a once weekly injectable, is the first long-acting amylin analogue developed for obesity treatment and has shown efficacious weight reduction with superiority to Liraglutide 3.0 mg at the higher dose [13, 14]. This has excitingly led to another combination therapy in the pipeline that utilises the alternate anti-obesity actions of amylin with the well-established class of GLP-1 mimetic; Cagrilintide with Semaglutide.

## Physiology of GIP

Alongside GLP-1, GIP is the other major incretin hormone produced in response to oral nutrient intake. GIP is secreted from the enteroendocrine K cells of the upper intestine and acts via the GIP receptor (GIPR) on pancreatic beta cells to directly promote glucose-stimulated insulin secretion. GIPR is also known to act on pancreatic alpha cells to potentiate amino acid-stimulated glucagon secretion as well as indirectly stimulating insulin secretion through paracrine actions [10].

GIPR is also expressed in white adipose tissue (WAT) and GIP may augment the ability of adipocytes to acutely clear dietary triglyceride, as well as improve the long-term storage of lipids through facilitating the healthy expansion of WAT which reduces ectopic fat accumulation in the liver, skeletal muscle, and pancreas [15].

GIPR has recently been identified in key feeding centres within the hypothalamus [16] with both acute and chronic central administration of acyl-GIP resulting in decreased body weight and food intake in mice. There is blunted/absent efficacy in knockout mice who have selective loss of hypothalamic GIPR suggesting a key role for hypothalamic GIPR in the control of energy metabolism [17]. Furthermore, GIPR is expressed in oligodendrocytes within the mediobasal hypothalamus [16] which regulate the access of peripheral signals to the arcuate nucleus [18]. This suggests a mechanism by which central GIP activity may potentiate the action of peripheral satiety signals such as GLP-1 by increasing its exposure to anorexigenic neuronal populations within the hypothalamus.

GIP exhibits both anti-atherogenic and pro-atherogenic properties in vitro. However, GIP infusion reduces mean arterial blood pressure by 10–15 mmHg, with < 8 bpm increase in mean heart rate in individuals with normal glucose tolerance, impaired glucose tolerance, and T2DM. This is in keeping with GLP-1 agonists which have shown similar effects on systolic blood pressure and heart rate, therefore it is likely that overall GIP is protective against atherosclerosis and cardiovascular disease [19].

GIP has received less attention compared to GLP-1 as a therapeutic target in obesity due to early findings that GIP physiologically stimulates glucagon secretion and lipogenesis. Moreover, loss of GIP secretion either through genetic, immunologic, or pharmacological means promotes weight loss, thus GIP has been traditionally perceived as an obesogenic hormone [10, 20]. However, recent data indicate both activation and inhibition of GIPR provide beneficial effects on body weight and metabolism [21] which has generated much debate regarding the receptor and postreceptor pathways involved in GIP signalling.

### GIP agonism

GIP infusions resulting in plasma concentrations similar to those observed following oral glucose ingestion, stimulate insulin secretion in a glucose-dependent manner with maximal efficacy at higher postprandial glucose levels, however, its effect is blunted in obesity and T2DM [20, 22]. GIP infusion has been shown to promote storage of dietary triglycerides in WAT and reduce circulating free fatty acids in patients with T2DM [15].

Mroz et al. utilised a series of structurally diverse GIP agonists to assess in vitro and in vivo effects on body weight and glycaemic control in obese mice. They found that chronic daily treatment with GIP agonists led to consistent dose-dependent body weight reduction without alterations in energy expenditure. The weight-lowering effect was preserved in GLP-1 receptor knockout mice but absent in GIPR knockout mice confirming selectivity towards GIPR. The body weight-lowering efficacy was more pronounced with GIP agonists that were optimised for GIPR selectivity, cross-species activity and duration of action [23].

Daily treatment with the GIP agonist ZP4165 did not significantly affect body weight and food intake in mice, however, co-administration of ZP4165 with the GLP-1 agonist liraglutide led to weight loss and food intake reduction that was significantly greater than treatment with liraglutide alone suggesting a synergistic effect [24].

Subsequently, GIP/GLP-1 co-agonism has engendered much interest, with the first dual GIP/GLP-1 co-agonist NN9709 (formerly MAR709 and RG7697) able to improve glycaemic control and reduce body weight compared to placebo in phase 2 clinical trials. However, its effects were not superior to liraglutide alone [25].

### GIP Antagonism

In mouse studies, a long-acting GIP analogue with high in vitro antagonism potency for GIPR was able to acutely inhibit the effects of GIP to improve glucose tolerance. However, chronic treatment with high doses of this antagonist did not result in significant body weight change [23]. Recently, studies using a monoclonal antibody-based GIPR antagonist (GIPR-Ab) both alone and in combination with GLP-1, demonstrated effective suppression of body weight rise in obese mice and non-human primates [26, 27]. Furthermore, weight loss was greater with GIPR-Ab/GLP-1 bispecific molecules than with GIPR antagonist alone, suggesting synergistic effects.

When results are compared across studies, it seems that GIP agonism and antagonism produce similar changes in body weight and both agonists and antagonists achieve synergistic weight loss when combined with GLP-1 agonists [20]. The molecular pathways underlying these effects have not been fully elucidated, however, possible unifying mechanisms include agonist-induced GIPR desensitization [28] and changes in receptor internalisation processes in response to prolonged agonist time may downregulate the GIP system to the extent that it mimics antagonism [29].

Both the GIP/GLP-1 co-agonists; NN9709 and Tirzepatide induce unique spatiotemporal GIP and GLP-1 receptor signalling, trafficking, and recycling dynamics compared to native peptides, Semaglutide, and matched mono-agonist controls. These findings support the hypothesis that the structure of GIP/GLP-1 co-agonists confer a biassed agonism that, in addition to its influence on intracellular signalling, uniquely modulates receptor trafficking [30]. While GIP/GLP-1 co-agonism using Tirzepatide shows very promising results in clinical trials, there have been few studies evaluating GIP antagonism alone and in combination with GLP-1 in humans [31, 32].

## Tirzepatide

Tirzepatide (formerly LY3298176) is a synthetic molecule containing 39 amino acids with dual functionality that targets both the GLP-1 and GIP receptor [33]. It is administered subcutaneously as a once weekly regimen with a prolonged half-life of five days and fivefold increased potency at the GLP-1 receptor [1, 34]. Clinical data from preliminary studies promisingly demonstrated an enhanced incretin effect with the synergistic co-activation of GIP and GLP-1 receptors when dually targeted in obese mice as well as T2DM subjects [11, 25, 35]. Thus, Tirzepatide was developed on the potential basis that the metabolic action of GIP augments the clinical efficacy observed with GLP-1 receptor agonists and thereby can broaden therapeutic benefit in lowering glucose as well as body weight [34].

### Tirzepatide in phase I and II trials

A phase I placebo-controlled, double-blind and randomised control trial (RCT) conducted in healthy subjects as well as T2DM patients evaluated Tirzepatide in terms of safety, tolerability and clinically meaningful glycaemic and weight control [34]. Albeit limited by sample size as well as duration, the study design consisted of four-week treatment with a single ascending dose (SAD) and multiple-ascending dose (MAD) protocols in healthy subjects followed by a multiple-dose proof-of-concept (POC) in T2DM patients [34]. The 4-week MAD study tested four arms of weekly Tirzepatide (0.5 mg, 1.5 mg, 4.5 mg or a dose-escalation to 10 mg) in comparison to placebo as well as the selective GLP-1 receptor, Dulaglutide 1.5 mg weekly. By day 29, Tirzepatide treatment demonstrated a dose-dependent weight loss that was significantly greater than placebo at all doses except for 0.5 mg with a reduction from baseline of − 4.52 kg and − 4.05 kg in the 4.5 mg and 10 mg groups, respectively (vs − 1.3 kg for Dulaglutide) [36]. The phase 1b POC study in T2DM tested two fixed doses (0.5 mg and 5 mg Tirzepatide) as well as two dose-titration schedules to 10 mg (5/5/10/10 mg Tirzepatide) and 15 mg (5/5/10/15 mg Tirzepatide) over a 28-day treatment period [34]. In addition to glycaemic efficacy, Tirzepatide treatment similarly demonstrated body weight reduction in a dose-related manner with a mean change from baseline of − 2.39 kg and − 2.95 kg in the 10 mg and 15 mg groups, respectively (vs − 0.32 kg in placebo) [36].

Another phase I MAD study in 48 Japanese T2DM participants evaluated one fixed dose (5 mg) and two dose-titrations (2.5 mg for 2 weeks, 5 mg for 2 weeks and 10 mg for 4 weeks or 5 mg for 2 weeks, 10 mg for 4 weeks and 15 mg for 2 weeks) treatment groups of Tirzepatide over an 8-week exposure period [37]. From week 5, Tirzepatide treatment resulted in body weight reduction from baseline in a dose-dependent manner with a mean loss of − 3.4 kg, − 5.0 kg and − 6.6 kg in the 5 mg, 10 mg and 15 mg treatment groups, respectively (vs a gain of 1.46 kg with placebo) [37]. Glycaemic efficacy was likewise evident in the Tirzepatide-treated groups with statistically significant reductions in fasting plasma glucose and HbA1c level [37]. Interestingly, by week 8, Tirzepatide led to a dose-related decrease in meal consumption as well as a quantitatively measured reduction in hunger and fullness scores as per analysis of appetite ratings by a visual analogue scale [37]. Almost half of Tirzepatide-treated patients experienced a reduced appetite not associated with nausea or vomiting and ≥ 50% of meal leftover occurred in 13.6, 18.2 and 30.0% of the 5 mg, 10 mg and 15 mg treatment groups, respectively, by day 51 (vs 0% in placebo group) [38].

A phase 2 RCT was performed across 47 sites in 318 T2DM participants (aged 18–75) with a BMI of 23–50 kg/m^2^ and glycated haemoglobin (HbA1c) of 7.0–10.5%, who had suboptimal control through lifestyle intervention alone or stable metformin monotherapy [39]. Tirzepatide was evaluated at various doses of 1 mg, 5 mg, 10 mg and 15 mg compared to placebo as well as Dulaglutide 1.5 mg. The use of Tirzepatide reduced body weight as well as HbA1c in a dose-dependent manner against placebo with the highest dose of 15 mg achieving a mean weight loss of approximately 12% after 26 weeks treatment [39, 40]. Relative to Dulaglutide, Tirzepatide comparatively attained a greater degree of weight loss and HbA1c reduction at doses of 5 mg, 10 mg and 15 mg. The mean weight change was − 0.9 kg, − 4.8 kg, − 8.7 kg and − 11.3 kg from baseline in the 1 mg, 5 mg, 10 mg and 15 mg Tirzepatide-treated groups, respectively (vs − 0.4 kg placebo and − 2.7 kg Dulaglutide) [33, 39].

In addition, more participants treated at this dose range (Tirzepatide 5 mg, 10 mg and 15 mg) reached clinically meaningful body weight loss targets than those on dulaglutide or placebo at 26 weeks. 14–71% of Tirzepatide-treated patients had lost ≥ 5% body weight (vs 0% placebo and 22% Dulaglutide) and 6–39% lost ≥ 10% (vs 0% placebo and 9% Dulaglutide) [39]. Remarkably, 21.6 and 24.5% in the 10 mg and 15 mg Tirzepatide groups, respectively, achieved more than 15% body weight loss [39]. The potency of Tirzepatide in body weight reduction was echoed in its glycaemic efficacy; approximately one fifth and one third of the 10 mg and 15 mg Tirzepatide-treated groups achieved normoglycaemia (indicated by an HbA1c < 5.7%) [39].

A subsequent phase 2 trial by Frias et al. evaluated three dose-escalation regimens of Tirzepatide in T2DM patients; 12 mg (4 mg weeks 0–3; 8 mg weeks 4–7; 12 mg weeks 8–11), 15 mg-1 (2.5 mg weeks 0–1; 5 mg weeks 2–3; 10 mg weeks 4–7) and 15 mg-2 groups (2.5 mg weeks 0–3; 7.5 mg weeks 4–7; 15 mg weeks 8–11) [41]. The data echoed the efficacy of the prior phase 2 study with statistically significant HbA1c reduction from baseline at 12 weeks as well as dose-dependent weight loss that ranged from − 5.3 to − 5.7 kg in the Tirzepatide groups (vs − 0.5 kg placebo) [41]. Moreover, the study demonstrated that initiation of Tirzepatide at a lower starting dose and with smaller increments during the escalation period led to improved discontinuation rates as well as a lower incidence of nausea, vomiting and diarrhoea [41]. Comparable to a GLP-1 receptor analogue, the side effect profile of Tirzepatide is largely gastrointestinal in nature with nausea, vomiting and diarrhoea as well as decreased appetite being the most frequently observed events [33, 41]. The incidence of gastrointestinal events increased in a dose-dependent manner, were predominantly mild to moderate in severity and usually transient [39]. A prolonged escalation regimen thereby enables maximum dose efficacy whilst achieving a more favourable side effect profile; a key consideration for application in phase 3 development.

### The SURPASS clinical trials

The efficacy and safety of Tirzepatide has been under evaluation for therapeutic use in T2DM through a series of Phase 3 studies; the SURPASS clinical trials. The primary outcome for these trials is change from baseline in HbA1c level, aside from SURPASS-J combo and SURPASS-CVOT which are principally aimed around serious adverse events and cardiovascular outcomes, respectively [42, 43]. The trials include T2DM participants treated with lifestyle intervention alone (SURPASS-1, SURPASS-J mono) or oral agents in the form of Metformin, Sulfonylurea, Pioglitazone, SGLT2-inhibitor (SURPASS-2, SURPASS-3, SURPASS-4, SURPASS-J combo, SURPASS-AP combo) and/or insulin (SURPASS-5, SURPASS-6) [43–51]. They encompass either a placebo-controlled study design (SURPASS-1, SURPASS-5) or the use of active comparators; GLP-1 RA drugs [Dulaglutide (SURPASS J-mono, SURPASS-CVOT) and Semaglutide (SURPASS-2)] or short- and long-acting insulin analogues [Glargine (SURPASS-AP combo, SURPASS-4), Degludec (SURPASS-3) and lispro (SURPASS-6)] [33, 42, 44–47, 49–51]. Based on results from the Phase 2 trials, a dose-escalation algorithm for Tirzepatide is utilised in the Phase 3 SURPASS studies with an incremental increase of 2.5 mg every 4 weeks (from an initial dose of 2.5 mg) until a maintenance of once weekly 5 mg, 10 mg or 15 mg is reached.

SURPASS-1 is the first RCT of Tirzepatide that demonstrated both a progressive and dose-dependent weight reduction in participants with T2DM that was inadequately controlled by diet and exercise alone, in addition to potent glucose-lowering [44]. Tirzepatide induced a weight change from baseline of − 7.0 kg, − 7.8 kg and − 9.5 kg in the 5 mg, 10 mg, and 15 mg groups, respectively (vs − 0.7 kg placebo), and the reduction was observed by week 4 but did not plateau by week 40 [44]. Furthermore, a significantly greater proportion of Tirzepatide-treated patients reached weight loss of ≥ 5% (67–78%), ≥ 10% (31–47%) or ≥ 15% (13–27%) compared to 14, 1 and 0% of participants in the placebo group, respectively [44].

Another 40-week trial (SURPASS-2) in 1879 T2DM patients on metformin monotherapy further demonstrated that Tirzepatide at all doses was superior to the GLP-1 RA, Semaglutide 1 mg once weekly, in reducing HbA1c as well weight from baseline [47]. The mean reduction in body weight was − 7.6 kg, − 9.3 kg and − 11.2 kg with 5 mg, 10 mg and 15 mg Tirzepatide doses, respectively, versus − 5.7 kg for Semaglutide at 40 weeks [47]. More participants achieved greater magnitudes of weight loss in the Tirzepatide-treated group; 65–80% lost ≥ 5% body weight (vs 54% Semaglutide), 34–57% lost ≥ 10% (vs 24% Semaglutide) and 15–36% had body weight reduction of at least 15% (vs 8% Semaglutide) [47]. Another study that utilises GLP-1 RA as a comparator is SURPASS-J mono which evaluates Tirzepatide versus weekly Dulaglutide 0.75 mg in T2DM participants [48]. Tirzepatide demonstrated a dose-related mean reduction in body weight of − 5.8 kg (− 7.8%), − 8.5 kg (− 11.0%) and − 10.7 kg (− 13.9%) for the 5 mg, 10 mg and 15 mg groups, respectively, compared to − 0.5 kg (− 0.7%) with Dulaglutide [48].

The profound weight loss efficacy of Tirzepatide versus placebo is further evident in T2DM patients on insulin treatment with titrated glargine in the 40-week SURPASS-5 trial. The mean change in body weight from baseline was − 5.4 kg, − 7.5 kg and − 8.8 kg in the 5 mg, 10 mg 15 mg Tirzepatide-treated groups, respectively, in stark contrast to the 1.6 kg weight gain observed in the placebo with glargine group [46]. Furthermore, 40 weeks of Tirzepatide treatment with glargine resulted in a significantly greater HbA1c reduction of 2.11–2.40% (vs 0.93% for placebo and glargine), indicating dual superiority in both glycaemic as well as weight loss efficacy [46]. Tirzepatide has consistently demonstrated improved diabetic control with the added benefit of weight loss to a robust magnitude in T2DM patients in further SURPASS studies published that utilised short- or long-acting insulin analogues as active comparators.

In SURPASS-3, Tirzepatide was superior to titrated insulin Degludec with greater reductions in HbA1c as well as body weight observed at week 52 in insulin-naïve T2DM patients treated with metformin alone or in combination with an SGLT2 inhibitor [45]. All three Tirzepatide doses induced weight loss at an average of 7.5–12.9 kg compared to a mean weight gain of 2.3 kg in the Degludec-treated group, and also significantly lowered the risk of hypoglycaemia [45]. The trial additionally observed that all three doses of Tirzepatide resulted in significantly greater reduction in liver fat content compared to insulin Degludec (− 29.78 to − 47.11% vs − 11.17, respectively) [52]. Given the crucial pathophysiology between adipose tissue and insulin resistance, these findings carry therapeutic implications for dual GIP and GLP-1 receptor agonism in targeting ectopic fat accumulation. The efficacy and safety of Tirzepatide in biopsy-proven non-alcoholic steatohepatitis is primarily being explored in the placebo-controlled phase 2 study SYNERGY-NASH [53].

### The SURMOUNT clinical trials

The SURMOUNT-1 trial importantly investigated the efficacy of Tirzepatide in the treatment of obesity and its ability to induce substantial as well as sustained weight loss in patients without T2DM [54]. The placebo-controlled RCT assigned 2539 participants of BMI ≥ 30 kg/m^2^ (or ≥ 27 kg/m^2^ with at least one weight-related comorbidity) to weekly Tirzepatide 5, 10 or 15 mg) with the primary endpoints being percentage change in weight and weight reduction of 5% or more [54]. During 72 weeks treatment, Tirzepatide-treated participants achieved an average weight loss from baseline of 15.0, 19.5 and 20.9% at doses of 5 mg, 10 mg and 15 mg, respectively, compared to merely 3.1% in the placebo group [54]. Weight loss of ≥ 5% was achieved by 85, 89 and 91% of participants treated with 5 mg, 10 mg and 15 mg of Tirzepatide, respectively (vs 35% placebo) [54]. Remarkably, 50% of the 10 mg and 57% of the 15 mg Tirzepatide groups lost at least 20% of their body weight compared to 3% with placebo [54]. Hence, a significantly higher percentage of Tirzepatide-treated participants not only achieved the primary endpoint of ≥ 5% weight loss than placebo but reached efficacy of even remarkably greater magnitude that is comparable to bariatric surgery.

Further studies underway include the SURMOUNT-2 trial which is evaluating the higher Tirzepatide doses of 10 mg and 15 mg in patients who also have T2DM [55]. SURMOUNT-3 is assessing whether Tirzepatide can enhance or maintain weight loss achieved through intensive lifestyle intervention, with similar primary endpoints of percentage change in body weight from baseline and weight reduction of 5% or more [56]. Finally, SURMOUNT-4 will assign SURMOUNT-3 participants who have had 36 weeks of Tirzepatide to continue treatment or switch to placebo and assess at 88 weeks whether they have lost, maintained, or regained weight loss from the point of randomisation [57]. Thus, the data from these trials will enable further evaluation in the sustainability of the efficacious weight loss observed with Tirzepatide treatment.

## Amylin-based therapies

Amylin, a 37-amino acid pancreatic hormone that is co-secreted with insulin, appears to exert a beneficial weight-lowering effect that is centrally mediated through appetite control and thus modulation of energy intake [58]. It has a recognised role in satiety signalling given its characteristics in reducing food ingestion, delaying gastric emptying, and suppressing glucagon secretion [59]. Thus, its endogenous anti-obesity effect as a satiating agent has led to the development of novel analogues that can be clinically utilised as such.

### Pramlintide

Given its incretin action, amylin also possesses anti-glycaemic effect through consequent suppression in glucagon secretion as well as hepatic glucose production and thus lowering of postprandial glucose levels [60, 61]. These glucose-lowering properties have been harnessed for clinical use in insulin-treated diabetic patients through the development of its short-acting and injectable synthetic analogue, Pramlintide. In patients with type 1 or 2 diabetes on insulin, studies have shown that the addition of Pramlintide leads to improved glycaemic parameters with a significantly greater HbA1c reduction that is accompanied by weight loss, rather than gain, and without increased risk of adverse hypoglycaemia [62–64]. Pramlintide-mediated reduction in postprandial hyperglycaemia as well as weight thereby offers a safe, effective, and metabolically favourable treatment escalation alternative for patients with suboptimal diabetic control that require intensification of insulin titration. In 2005, it received approval by the United States (US) Food and Drug Administration (FDA) as an adjunct to mealtime insulin therapy in patients with type 1 or 2 diabetes [61]. Albeit modest, its weight-lowering potential has also been explored. Pramlintide-treated obese subjects in a phase 2 dose-escalation study achieved placebo-corrected body weight reduction of 3.7 ± 0.5% (3.6 ± 0.6 kg) and approximately 31% experienced ≥ 5% weight loss [65]. Pramlintide’s anti-obesity capabilities extend beyond weight loss as it has also demonstrated reductions in 24 h caloric intake, portion size, fast food ingestion and improvement in binge eating behaviour [66].

### Cagrilintide

Several long-acting amylin analogues have thus far been evaluated in animal studies but only one in clinical trials; Cagrilintide is currently in phase 2 development and has demonstrated promising weight loss potential. A phase 2 RCT compared escalating doses of once weekly Cagrilintide (0.3, 0.6, 1.2, 2.4 or 4.5 mg) to lifestyle intervention or once daily liraglutide 3.0 mg in non-diabetic individuals of BMI ≥ 30 kg/m^2^ (or ≥ 27 kg/m^2^ with hypertension or dyslipidaemia) and had a primary endpoint of percentage change in body weight at 26 weeks [13]. Cagrilintide achieved a progressive as well as dose-dependent weight reduction from baseline, which did not plateau in the 26-week trial period and was superior at all doses to placebo (− 6.0%, − 6.8%, − 9.1%, − 9.7% and − 10.8% for Cagrilintide 0.3 mg, 0.6 mg, 1.2 mg, 2.4 mg and 4.5 mg, respectively vs -3.0% for placebo) [13]. At the highest dose of 4.5 mg, Cagrilintide demonstrated greater weight loss than the liraglutide group (− 10.8% vs − 9.0%, respectively) [13]. Of note, however, Liraglutide showed a reduction in HbA1c and fasting plasma glucose but there was no observed change in these parameters with Cagrilintide at any dose by week 26 [13].

### Cagrilintide plus semaglutide

Mechanistic insight thus far has shown how chronic exposure to amylin as well GLP-1, or their mimetics, leads to suppression of food intake and weight gain through diverse metabolic functions [67]. There is increasing appreciation around the stratagem of combination therapy to synergistically enhance the weight loss effect due to overlapping neuroendocrine mechanisms in control of energy balance and thereby body weight. Data from a study in diet-induced obese rats demonstrated that co-administration of amylin with GLP-1, or its receptor analogue, resulted in greater reduction of food ingestion and therefore weight gain compared to individual monotherapy [68]. Consequently, there are ongoing clinical trials evaluating the safety and tolerability as well as efficacy of Cagrilintide/Semaglutide as a combined treatment for obesity and in diabetes.

A 20 week multiple-ascending dose phase I study investigated the efficacy of once weekly Cagrilintide and 2.4 mg Semaglutide in participants with obesity with a BMI of 27.0 to 39.9 kg/m^2^ without lifestyle interventions [69]. The trial consisted of six cohorts where individuals were randomly assigned to a dose of Cagrilintide (0.16, 0.30, 0.60, 1.2, 2.4 or 4.5 mg) by once weekly subcutaneous injection combined with Semaglutide 2.4 mg, or matched placebo with Semaglutide 2.4 mg [69]. The primary outcome was the number of treatment-emergent adverse events from baseline to end of the follow-up period (week 25) and weight loss was evaluated as an exploratory endpoint. At 20 weeks, a clinically significant reduction in body weight was apparent across all treatment groups and particularly evident at the larger Cagrilintide doses in combination with Semaglutide. The mean percentage body weight reduction was as high as 17.1% (− 15.9 kg) for Cagrilinitide 2.4 mg and 15.7% for Cagrilinitide 1.2 mg compared to 9.8% in the pooled placebo group, all in combination with 2.4 mg Semaglutide [69]. The Cagrilinitide 4.5 mg/Semaglutide 2.4 mg combination achieved a mean weight loss from baseline of − 15.4% (− 15.0 kg) vs − 8.0% (− 7.8 kg) in the matched placebo/Semaglutide 2.4 mg group [69]. Gastrointestinal disorders were the most common side effect reported but mild to moderate in severity (nausea, vomiting and dyspepsia). Although the addition of Cagrilintide to Semaglutide increased gastrointestinal disorders, it was not contingent on Cagrilintide dose and only two participants discontinued treatment due to adverse events [69]. Individuals more frequently reported decreased appetite with the higher doses of Cagrilintide and early satiety with the 1.2 mg dose or higher of Cagrilintide, than placebo in combination with Semaglutide.

Despite these promising results, especially given that no lifestyle intervention was combined with pharmacotherapy, the single-centre study is limited by its short treatment duration as well as sample size and patient cohort (mean age 40.6 years with no comorbid diabetes or cardiovascular disease) [70]. Hence, the outcome of further studies is awaited for long-term drug evaluation of the Cagrilinitide/Semaglutide combination strategy to confirm safety, tolerability and weight-lowering efficacy in target populations.

## Setmelanotide

The leptin-melanocortin pathway plays a crucial role in the central control of food intake. Melanocyte-stimulating hormone, which is cleaved from the pro-hormone proopiomelanocortisin (POMC) is pivotal in the regulation of satiety and energy expenditure and mediates the anorectic effect of leptin via its action at the melanocortin-4 receptor (MC4R) [71].

Setmelanotide is a potent MC4R agonist which displays both clinical efficacy and minimal undesirable cardiovascular effects and has proven to be a highly promising new addition to the armamentarium for personalised obesity pharmacotherapy. The pivotal study by Kuhnen et al. demonstrated that Setmelanotide administration to patients with POMC deficiency resulted in major weight loss of 20.5 kg and 51.0 kg over 12 and 42 weeks respectively [72], highlighting the potent impact of the melanocortin pathway on bodyweight regulation. Subsequently a phase 3 trial which included individuals with POMC and Leptin receptor deficiency found that Setmelanotide significantly reduced body weight and hunger scores in both groups [73]. One phase IIb clinical trial administered Setmelanotide to participants with obesity with heterozygous MC4R deficiency and obese controls over 28 days, observing a placebo-adjusted weight loss of 2.6 kg and 4.0 kg respectively [74]. At present, Setmelanotide is approved by the US FDA and the United Kingdom National Institute for Health and Care Excellence (NICE) as a treatment for obesity and hyperphagia specifically caused by POMC deficiency, including proprotein convertase subtilisin/kexin type 1 or leptin receptor deficiency in people aged 6 years and over [71, 75]. Setmelanotide is also being investigated in other rare genetic disorders associated with obesity, and a phase 3 trial using Setmelanotide in Bardet-Beidl syndrome and Alström Syndrome is currently ongoing [76]. Although it seems that MC4R agonists have a small remit in the treatment of rare genetic causes of obesity, gaining a better understanding of the genetics of severe obesity has helped unveil the importance of the melanocortin pathway in appetitive control, and may pave the way for new avenues of obesity pharmacotherapy in the future.

## Emerging therapies

### Fibroblast growth factor 21

Fibroblast Growth Factor 21 (FGF21) is a circulating hormone induced in the fasting state and implicated in the metabolic response to fasting. Multiple studies using FGF21 and FGF21 analogues demonstrate robust control of glucose and body weight reduction in preclinical studies. Administration of the FGF21 analogue LY2405319 to humans with obesity and T2DM resulted in reduction in body weight but no significant change in glycaemia after 28 days; however, circulating levels of low-density lipoprotein cholesterol and triglycerides were reduced, whereas levels of high-density lipoprotein cholesterol were increased [77].

Considerable ongoing interest in FGF21 stems from its beneficial actions on hepatic steatosis. Preclinical studies investigating the long-acting FGF21 analogue, B1344, in non-human primates with non-alcoholic steatohepatitis (NASH) demonstrated reduction of steatosis, attenuation of inflammation and fibrosis, and reduced hepatocyte injury after 11 weeks of therapy [78]. B1344 also attenuated hepatic inflammation and liver injury in mice fed on a methionine- and choline-deficient diet. A long-acting FGF21 analogue, pegbelfermin, administered once a day at a dose of 10 or 20 mg for 16 weeks to overweight or obese humans with NASH reduced hepatic steatosis without changes in body weight or bone mineral density [79].

A unimolecular long-acting GLP-1/FGF21 co-agonist produced greater reduction of glycaemia and body weight relative to single agonists without evidence of fasting-associated hypoglycaemia [80]. Whether FGF co-agonists can be feasibly used as therapies in metabolic disorders without adverse effects remains unclear. Nevertheless, as both GLP-1 and FGF21 agonists are being closely studied as therapeutic avenues or NASH, it would be reasonable to consider a combined approach given their distinct mechanisms of action.

### Ghrelin antagonism

Ghrelin is the only known orexigenic peptide hormone and induces hunger by stimulating neuropeptide Y neurons and inhibiting hypothalamic POMC neurons. Inhibition of the ghrelin receptor represents an attractive therapeutic target in obesity. Ghrelin receptor antagonists and vaccines have shown promise with reduced food intake and body weight in pre-clinical studies [81, 82]. However, results in human trials are less favourable with no difference in weight between obese patients receiving ghrelin immunisation over 20 weeks [83]. More recently ghrelin receptor inverse agonists have been successfully profiled in humans with few adverse effects [84].

### GLP-1/glucagon co-agonists

Building on the success of the GIP/GLP-1 co-agonist Tirzepatide, GLP-1/Glucagon co-agonists that harness glucagon receptor activation to promote energy expenditure in addition to GLP-1 effects on reduced calorie intake hold great promise for synergistic weight loss. Furthermore, the naturally occurring gut hormone oxyntomodulin mediates its anorectic effect and increases energy expenditure through its actions at both the GLP-1 and glucagon receptor [85]. MEDI0382, a once daily GLP-1/glucagon co-agonist has been shown to significantly reduce body weight and improve glycaemic control in phase 2 trials with good tolerability [86]. Recently, a once weekly GLP-1/glucagon co-agonist, LY3305677 (IBI362) has completed phase 1 trials with reported mean bodyweight loss of 4.8–6.4% in overweight and obese adults [87] and improved glycaemic control in patients with T2DM [88].

### GLP-1/GIP/glucagon triple agonists

Following on from the exploration of GLP-1/glucagon co-agonists, it is hoped that the synergistic action of unimolecular GLP-1/GIP/Glucagon triple agonists will provide profound effects on weight loss and glucose metabolism. Two triple agonists (HM15211 and MAR423) are currently undergoing Phase 1 clinical trials with no published clinical trial data at present [89]. However, preclinical studies of these drugs demonstrate promising results in rodent models [90].

### Growth differentiation factor 15

Elevated circulating levels of growth differentiation factor 15 (GDF15) have been shown to reduce food intake and lower body weight through activation of hindbrain receptor glial-derived neurotrophic factor (GDNF) receptor alpha-like (GFRAL) in rodents and non-human primates. Evidence also suggests GDF15 may improve NASH through anti-inflammatory pathways and induce cardioprotective effects by reducing atherosclerosis, cardiac hypertrophy, and ischaemia–reperfusion injury. Thus, endogenous induction of this peptide holds promise for obesity treatments and clinical trials of long-acting GDF15 analogues are currently in progress [91, 92].

### Melanin-concentrating antagonists

Melanin-concentrating hormone (MCH) is a 19 amino acid long peptide found in the brains of several mammalian species and has been implicated in appetite control and energy homeostasis. GPS18169, a pseudopeptide antagonist at the MCH-R1 receptor has been shown to reduce adiposity and normalise insulin levels in mice fed a high fat diet with no change in food or water consumption [93].

## Conclusion/future directions

Given the encouraging results of the latest SURPASS and SURMOUNT trials, Tirzepatide appears to be the current frontrunner in future obesity pharmacotherapy. Combination therapy, however, is an auspicious strategy and the preliminary data for the use of Cagrilinitide/Semaglutide shows equal, if not superior, promise to Tirzepatide with a comparable safety profile. Albeit the favourable outcome of these latest trials, future work will need to address the impact of prolonged treatment on safety and tolerability as well as the consequences of cessation on weight regain. Moreover, with greater mechanistic understanding in the underlying neuroendocrinology, the direction of travel for weight management will likely become personalised to individual characteristics and co-morbidities. The best example of this precision medicine approach is the launch of Setmelanotide which causes very substantial weight loss in people with specific mutations of the hypothalamic appetite signalling system. The obesity pandemic is affecting a diversely complex patient population and needs an algorithmic approach that efficaciously utilises the distinct profile of each drug class that becomes available.

## Data Availability

Not Applicable.
